# Supplementary material for: Temporal trends in aerobic physical activity guideline adherence among nationally representative samples of U.S adults between 2011 and 2019: Cross-sectional findings from a sample of over 2 million adults
Source: PLoS One. 2025 Jan 8;20(1):e0316051. doi: 10.1371/journal.pone.0316051 (PMC11709288; doi:10.1371/journal.pone.0316051)
Supplement: S1 Table — (DOCX) [file pone.0316051.s001.docx]

**S Table 1: Seasonality Distribution of Conducted BRFSS Interviews for 2011, 2013, 2015, 2017 and 2019 Surveys**

| **Survey Year** | | | | | |
| --- | --- | --- | --- | --- | --- |
| **Interview Season**^a^ | **2011**  %^b^ (95% CI) | **2013**  %^b^ (95% CI) | **2015** %^b^ (95% CI) | **2017** %^b^ (95% CI) | **2019** %^b^ (95% CI) |
| Winter | 21.4 (21.2-21.7) | 22.8 (22.5-23.0) | 26.2 (25.9-26.5) | 21.5 (21.3-21.8) | 20.3 (20.0-20.5) |
| Spring | 26.9 (26.7-27.2) | 26.8 (26.6-27.1) | 25.4 (25.1-25.6) | 22.6 (22.3-22.8) | 26.7 (26.4-26.9) |
| Summer | 26.2 (26.0-26.5) | 25.5 (25.3-25.8) | 25.0 (24.7-25.2) | 29.9 (29.6-30.2) | 27.5 (27.2-27.8) |
| Autumn | 25.4 (25.1-25.7) | 24.8 (24.6-25.1) | 23.4 (23.2-23.7) | 26.0 (25.7-26.3) | 25.6 (25.3-25.8) |
| ^a^Winter = December, January and February, Spring = March, April and May, Summer = June, July and August, Autumn = September, October and November  ^b^Data weighted using stratum weight provided by the Centers for Disease Control and Prevention (CDC). | | | | | |
