# Supplementary material for: Temporal trends in aerobic physical activity guideline adherence among nationally representative samples of U.S adults between 2011 and 2019: Cross-sectional findings from a sample of over 2 million adults
Source: PLoS One. 2025 Jan 8;20(1):e0316051. doi: 10.1371/journal.pone.0316051 (PMC11709288; doi:10.1371/journal.pone.0316051)
Supplement: Attachment — Submitted filename: Abernethy_David_PLOS_One_Response to Reviewers.docx [file pone.0316051.s002.docx]

**Response Letter for PLOS ONE Manuscript [PONE-D-24-25566]**

Dear Dr Mahamat Fayiz Abakar,

We appreciate the time and effort that you invested in providing valuable feedback on our manuscript, *"Temporal Trends in Aerobic Physical Activity Guideline Adherence Among Nationally Representative Samples of U.S Adults Between 2011 and 2019: Cross-sectional Findings from a Sample of Over 2 Million Adults"* [PONE-D-24-25566]. We have carefully considered your suggestions and have responded to each point in detail below.

Reviewer Comments:

"I was wondering why the seasonality effect was not among the factors / variables assessed by the study. Were all the surveys conducted at the same period each year or not? As the main question was to answer based on what happened last month?

The point here is, if these surveys were conducted in different periods of the year, i.e. summer, winter, fall, etc. then there might be a bias that need to be adjusted for, at least statistically. Presenting a table indicating when exactly (month/season) the surveys were conducted each year would give an insight of this aspect.

Finally, considering this aspect as a one of the weakness of the study in the limitation section would be important.”

We appreciate your insightful comment regarding the potential influence of seasonality on physical activity behaviors. We acknowledge that seasonality can impact physical activity, as environmental factors like temperature and daylight availability may influence individuals’ capacity or motivation to engage in outdoor physical activity.

However, seasonality has not historically been accounted for in studies using BRFSS data. The timing of the surveys is spread across the year, likely resulting in seasonal fluctuations averaging out over such a large sample. Furthermore, adjusting for seasonality in our study focused on long term trends could shift the focus towards more cyclical fluctuations that are not relevant to our study’s primary research questions and potentially dilute the interpretations of long-term population activity adherence.

To address your concern, we have added a supplementary table (S1 Table in the revised manuscript) outlining the seasonal distribution of when the BRFSS survey interviews were conducted to provide readers with some insight into this aspect. The distribution of the survey interviews was relatively similar across the seasons for all BRFSS surveys used in our study. Additionally, we have added a statement to the limitations section acknowledging that seasonality may influence activity levels of individuals and to take this into account when interpreting the study’s results.

We sincerely thank you again for your careful consideration and valuable feedback. We hope that the revisions we have made address your concerns adequately. We believe that these changes further enhance the rigor and clarity of our manuscript, and we look forward to your final evaluation.

Sincerely,
David Abernethy
On behalf of all co-authors
